# Immunization with *Brugia malayi* Myosin as Heterologous DNA Prime Protein Boost Induces Protective Immunity against *B*. *malayi* Infection in *Mastomys coucha*

**DOI:** 10.1371/journal.pone.0164991

**Published:** 2016-11-09

**Authors:** Jyoti Gupta, Sweta Misra, Shailja Misra-Bhattacharya

**Affiliations:** 1 Division of Parasitology, CSIR-Central Drug Research Institute, BS 10/1, Sector 10, Jankipuram Extension, Sitapur Road, Lucknow-226031, India; 2 Academy of Scientific and Innovative Research, New Delhi, India; Midwestern University, UNITED STATES

## Abstract

The current control strategies employing chemotherapy with diethylcarbamazine, ivermectin and albendazole have reduced transmission in some filaria-endemic areas, there is growing interest for complementary approaches, such as vaccines especially in light of threat of parasite developing resistance to mainstay drugs. We earlier demonstrated recombinant heavy chain myosin of *B*. *malayi* (Bm-Myo) as a potent vaccine candidate whose efficacy was enhanced by heterologous DNA prime/protein boost (Myo-pcD+Bm-Myo) vaccination in BALB/c mice. BALB/c mouse though does not support the full developmental cycle of *B*. *malayi*, however, the degree of protection may be studied in terms of transformation of challenged infective larvae (L3) to next stage (L4) with an ease of delineating the generated immunological response of host. In the current investigation, DNA vaccination with Bm-Myo was therefore undertaken in susceptible rodent host, *Mastomys coucha* (*M*. *coucha*) which sustains the challenged L3 and facilitates their further development to sexually mature adult parasites with patent microfilaraemia. Immunization schedule consisted of Myo-pcD and Myo-pcD+Bm-Myo followed by *B*. *malayi* L3 challenge and the degree of protection was evaluated by observing microfilaraemia as well as adult worm establishment. Myo-pcD+Bm-Myo immunized animals not only developed 78.5% reduced blood microfilarial density but also decreased adult worm establishment by 75.3%. In addition, 75.4% of the recovered live females revealed sterilization over those of respective control animals. Myo-pcD+Bm-Myo triggered higher production of specific IgG and its isotypes which induced marked cellular adhesion and cytotoxicity (ADCC) to microfilariae (mf) and L3 *in vitro*. Both Th1 and Th2 cytokines were significantly up-regulated displaying a mixed immune response conferring considerable protection against *B*. *malayi* establishment by engendering a long-lasting effective immune response and therefore emerges as a potential vaccination method against LF.

## Introduction

Lymphatic filariasis (LF) is one of the most morbid and debilitating parasitic disease caused by *Wuchereria bancrofti*, *Brugia malayi* and *B*. *timori*. According to current estimates about 1.23 billion people in 58 countries worldwide are threatened by the disease, 120 million are infected and approximately 40 million remain disfigured and incapacitated. *B*. *malayi* possesses a complex life cycle and engenders a complicated host immune response with varied clinical manifestations [1].

To control this disease, no vaccine is in existence till date, however, a number of *B*. *malayi* proteins have been identified as vaccine candidates by us as well as other groups [2–9]. We earlier identified *B*. *malayi* recombinant heavy chain myosin (Bm-Myo) as potential prophylactic antifilarial vaccine candidate in a single and cocktail vaccine formulation [4, 10]. DNA based vaccines are relatively simple, inexpensive to produce [11] and have been shown to confer effective protection against several pathogens by inducing humoral and cellular immune responses in the immunized host. Filarial proteins such as chitinase[12], paramyosin[13], glutathione-S-transferase [14], tropomyosin [15], OvB20 [15], ALT-2 [16] and SXP-1 [16] have successfully been probed as experimental DNA vaccines. However, *in vivo* efficacy of DNA vaccine has not always been satisfactory. Attempts were made to enhance immunity by boosting with protein antigen that resulted in the generation of potent humoral and cellular immune responses that led to higher level of protection in veterinary and human infections [17, 18].

In heterologous prime boost, antigens are presented in different conditions that can enlarge the host immune response by increased activation of non-specific and specific co-stimulatory responses by the vector, giving a favorable milieu for antigen presentation. By priming with DNA plasmid and subsequent boosting with the protein, the antigen is processed and presented by antigen presenting cells via MHC class I or class II to trigger both humoral and cellular immune response [18–20].

In our previous work, homologous DNA (Myo-pcD) as well as heterologous DNA prime/protein boost (Myo-pcD+Bm-Myo) immunization in murine model (BALB/c) revealed generation of an effective Th1 and Th2 immune response that provided substantial protection against *B*. *malayi* larval (L3) challenge [21]. Filarial parasites are known to down-regulate the host immune response and inhibit antigen presentation for their survival and therefore protective immunity to filarial infections requires co-ordination of both Th1 and Th2 type of responses [22]. BALB/c mice support only preliminary development of challenged L3 to L4 or L5 stage but don’t support the full development cycle of *B*.*malayi*. Therefore, the current study performed DNA vaccination in permissive host, *Mastomys coucha* (*M*. *coucha*) with Bm-Myo employing recombinant protein as booster doses and effective long term protection was achieved against homologous larval challenge. *M*. *coucha* normally sustains the challenged L3 and facilitate their further development to sexually mature adult parasites with patent microfilaraemia[23].

## Materials and Methods

### Parasite–host

In the present study, 6 weeks old male *M*. *coucha* were maintained under appropriate housing conditions at Laboratory Animal Division of our Institute. All the animals and experimental protocols involving animal handling were duly approved by Institutional Animal Ethics Committee (IAEC) of Central Drug Research Institute(CDRI) constituted under the provisions of CPCSEA (Committee for the Purpose of Control and Supervision of Experiments on Animals), Government of India. The study bears approval no. IAEC/2011/120/Renew-3(111/15)/Dated-23/07/2014. To assess the animal health, all the animals were monitored daily, their body weights were taken, their body coat and alertness were observed on regular interval of one week.

*B*. *malayi* L3 for challenge experiments were recovered from the laboratory bred vector mosquitoes (*Aedes aegypti*) fed on donor *M*. *coucha* 9 ±1 day back [4].

### Preparation of plasmid construct (Myo-pcD)

The recombinant plasmid pcDNA3.1-Myo (Myo-pcD) was constructed as described earlier [21]. In brief, *bm-myo* (accession number AY705730) gene was cloned in mammalian expression vector pcDNA 3.1(+) (Invitrogen, USA) at *BamHI/XhoI* restriction sites and plasmid was isolated by Endo-free plasmid isolation kit (Qiagen, Germany) for immunizing *M*. *coucha*.

### Purification of *B*. *malayi* recombinant myosin protein (Bm-Myo)

The recombinant protein was expressed as described earlier [10]. In brief, *bm-myo* gene was cloned into expression vector pET 28a which was transformed in competent BL21 (DE3) *E*. *coli* cells. The recombinant protein was purified through Ni-NTA column and dialyzed to remove salts. The endotoxin level of purified protein was determined by LAL Chromogenic Endotoxin Quantitation Kit (Thermoscientific, USA) following manufacturer’s protocol.

### Immunization regimens

*M*. *coucha* were immunized as per the schedule described earlier [21]. In brief, sixty male *M*. *coucha* were divided to 6 groups containing 10 animals each, which were immunized on days 0, 15, 30 and 45. Out of the six groups, three groups (i, ii, iii) were kept as control groups for group iv, v and vi respectively. Animals of group i received all the doses of 100μg only pcDNA plasmid by intradermal (i.d) route, group ii were injected with Freund’s complete adjuvant (FCA) on day 0 followed by Freund’s incomplete adjuvant (FIA) on day 15, 30 and 45 by subcutaneous route (s.c) and the animals of group iii received pcDNA plasmid (on day 0 and 15) followed by FIA on day 30 and 45. The remaining three groups (iv, v, vi) were experimental groups. Group iv received Myo-pcD (100 μg plasmid construct by i.d route), animals of group v were administered with Bm-Myo (25 μg recombinant myosin protein emulsified in FCA/FIAon day 0/day 15, 30 and 45 by s.c. route, group vi was injected with Myo-pcD+Bm-Myo (100 μg Myo-pcD plasmid construct by i.d route on day 0, 15 followed by 25 μg Bm-Myo protein+ FIA on day 30 and 45, s.c). After two weeks of last booster, five *M*. *coucha* from each group were randomly euthanized by overdosing of intraval sodium (100 mg/kg) to measure cytokine mRNA expression in spleen cells while remaining animals (5 per group) were challenged subcutaneously with 100 *B*. *malayi* L3. The parasitological and immunological studies were undertaken in these two separate experiments. To lower the animal suffering, animals were anaesthetized by Ketamine (50 mg/kg) before blood collection. For each immunizing dose contained 100 μl volume which was injected at two separate spots in the back region using fine hypodermic needles causing minimal stress.

### Bm-Myo specific serum IgG and antibody isotype by ELISA

Blood for serum was collected from *M*. *coucha* before L3 challenge and thereafter at monthly intervals. Serum IgG antibody was measured on days 0, 16, 31, 46 and 60 since initiation of immunization and on days 30, 90, 120 and 180 post larval challenge (p.c) by indirect ELISA as described earlier [4, 23]. In brief, ELISA plates (Nunc, Denmark) were coated with Bm-Myo (1 μg/ml), blocked and reacted with Bm-Myo immunized *M*. *coucha* serum as the primary antibody (1:400) and rabbit anti-mouse-IgG-HRP (1:10000) as the secondary antibody (Sigma, USA). Absorbance was read at 492 nm by ELISA reader (Tecan, Switzerland) after adding the substrate orthophenyldiamine (OPD, Sigma) and stopping reaction with 2.5 M H_2_SO_4_.

Antibody isotypes were determined by antibody isotyping kit following manufacturer’s protocol (Sigma, USA) as described earlier [4]. Briefly, plates were coated with recombinant myosin (0.1 μg/ml), blocked, reacted with the serum samples collected before L3 challenge and on days 30, 90 and 180 p.c. Goat anti-mouse IgG1, IgG2a, IgG2b and IgG3 (1:1000) were used as secondary antibodies. Bound isotype specific antibodies were detected after adding HRP conjugated rabbit anti-goat IgG as mentioned above.

### Assessment of microfilaraemia, worm recovery and female worm fecundity

Monitoring of microfilaraemia was initiated in *M*. *coucha* from day 90 p.c and continued at monthly intervals up to day 180 by preparing thick blood smears as reported earlier [23, 24]. Briefly, 10 μl tail blood smears were prepared and stained with Leishman and microfilariae (mf) were counted microscopically. On day 180 p.c, animals were euthanized and adult worms were isolated from various tissues (lungs, heart, testes and lymph nodes) and counted. Arithmetic means were calculated for the total worm burden and percentage protection was calculated as [(u-v)/u x 100], where ‘u’ is the mean value for the control group and ‘v’ is the mean value for the experimental group. Each live female worm was teased on glass slide in a drop of PBS and intrauterine contents such as eggs, embryos and mf were observed microscopically to assess the effect of immunization on worm fecundity. Females having distorted eggs, degenerated embryos, dead or distorted mf or absence of mf was considered sterilized. Percentage sterilization was calculated as the ‘number of sterile female worms/total female worms teased x100’.

### Antibody-dependent cellular adhesion and cytotoxicity (ADCC) *in vitro*

To determine the role of antibodies in parasite killing, ADCC assay was performed as described earlier [4]. Briefly, 100 mf and ~10 L3 each were co-cultured at 37°C with 1×10^6^ PECs from naive *M*. *coucha* in 96 well plate in presence of sera collected from animals of different control and immunized groups (optimized dilution 1:16). After 48 h, cellular adhesion and cytotoxicity to mf and L3 was observed microscopically. Cellular adherence was considered positive if >20 cells and >5 cells were attached to surface of mf and L3 respectively, while limpid, damaged or immobile L3 were considered as dead.

Percentage cytotoxicity = Number of dead larvae ÷Total number of larvae × 100.

### Cytokine mRNA expression in spleen post-immunization and L3 challenge

The mRNA expression of Th1 (IL-2, IFN-ƴ, TNF-α, IL-12) and Th2 (IL-4, IL-10) cytokines in immunized and control mouse spleens were quantitated by qRT-PCR using custom designed gene-specific primers utilizing Beacon designer software. Mouse β-actin was used as an endogenous control gene. In brief, splenic tissues were homogenized in Trizol reagent and RNA was extracted as described earlier [25]. First strand cDNA was generated using Super Script III first strand cDNA synthesis kit (Invitrogen, USA) using oligo (dT) 20 primers. The specific cDNA fragments were amplified using SYBR premix and reaction was run on Roche applied System (Roche, US) under PCR conditions; 95°C for 5 min, followed by 40 cycles of 95°C for 20 s, 56°C for 15 s, and 72°C for 30 s. Relative amount of target amplicon in each experiment was determined by comparative ΔCT method [26].

### Statistical analysis

The data were analyzed by PRISM software (version 5.0) using one-way analysis of variance (ANOVA). Two-way ANOVA was employed wherever required and individual comparisons were made by Bonferroni method. Probability values (P) of < 0.05, < 0.01 and < 0.001 between immunized and control groups were considered as significant (*), highly significant (**) and very highly significant (***) respectively while P > 0.05 was considered as statistically non-significant.

## Results

All the immunized and control animals appeared active, healthy and showed a consistent increase in their body weight (data not shown). There was no mortality in any group of animals during the course study.

The endotoxin level of the recombinant purified protein preparation was found to be 0.089 EU/μg.

### Bm-Myo specific IgG antibodies increase considerably after DNA prime-protein boost immunization

**Fig 1:**
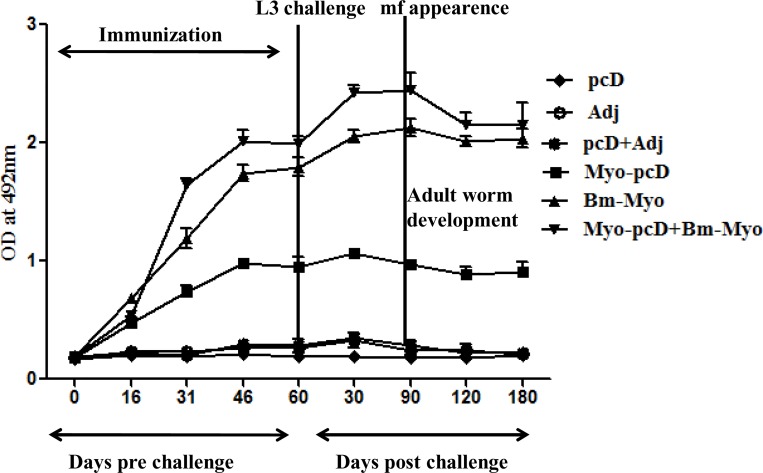
Anti-Bm-Myo antibody level measurement: Anti-Bm-Myo antibody levels were measured in the sera of all controls and immunized *M*.*coucha* before and after L3 challenge by ELISA. High Bm-Myo specific IgG titer was generated most significantly in Myo-pcD+Bm-Myo immunized animals compared to Bm-Myo(P < 0.05) and Myo-pcD (P < 0.001) that further increased after L3 challenge which slightly diminished after 90 days(mf appearance) p.c, however, remained high throughout the observation period.

Myo-pcD+Bm-Myo immunized animals produced significantly high titers of specific IgG antibody as compared to Bm-Myo and Myo-pcD group (P < 0.05; P < 0.001). Each antigen booster led to successive increase in the antibody level which increased further upon L3 challenge. IgG level showed a decline once mf started appearing in the tail blood though significantly elevated levels persisted till day 180 p.c (Fig 1). Nevertheless, Bm-Myo or Myo-pcD exhibited enhanced IgG titres (P < 0.001) over respective control group.

**Fig 2:**
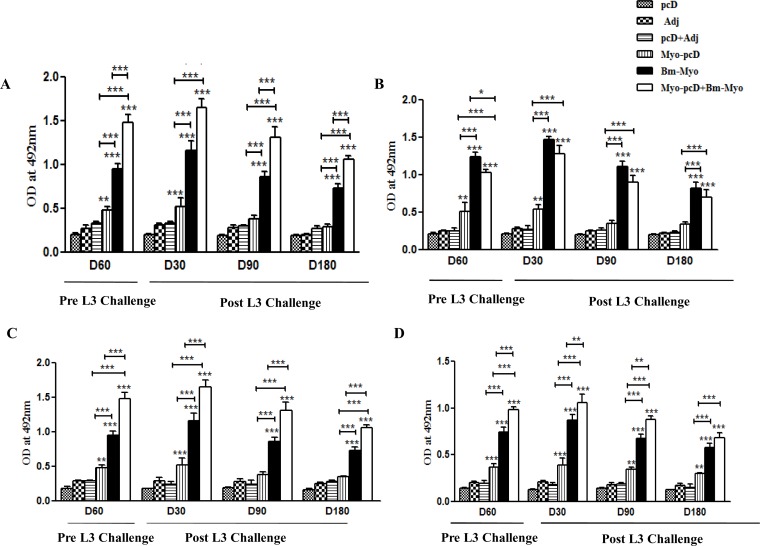
IgG isotypes antibody levels in *M*. *coucha*: IgG isotypes antibody levels were measured at after immunization (day 60) and 30, 90 and 180 day p.cin all the immunized and control groups. (A) High levels of IgG1, (B) IgG2a, (C) IgG2b and (D) Ig3 antibodies were found at all time points. Bars represent the mean±SE (n = 10) OD values (at 492nm). The statistical significance based on the differences between the mean values of immunized and control groups are indicated as *P < 0.05; **P < 0.01 and ***P < 0.001. Asterisks (*) on top of the bars represents statistical significance with their respective control group and above a line showed statistical significance with respect to each experimental immunized group.

The IgG isotypes were also analyzed by ELISA after last booster and subsequently on days 30, 90 and 180 p.c. All the three vaccine regimens triggered significant (P < 0.05–0.001) generation of specific IgG1, IgG2a, IgG2b and IgG3 post-immunization as compared to their respective control groups indicating a mixed Th1/Th2 type of response. On the other hand, both Myo-pcD+Bm-Myo or Bm-Myo groups demonstrated robust production of isotypes (P < 0.001), however IgG1:IgG2a ratio differed among these two groups, being >1 in prime boost immunization suggesting Th2 polarization while Bm-Myo revealed <1 ratio indicating Th1 bias. The data thus demonstrated that challenge with L3 (day 30) enhanced the level of IgG isotypes in the blood of *M*. *coucha* which declined between day 90 and 180, however, Myo-pcD+Bm-Myo vaccinated animals showed significantly elevated levels (P < 0.05–0.001) of IgG1, IgG2b and IgG3 till the day of autopsy(180 p.c) (Fig 2).

### Anti Bm-Myo antibody induces adherence of PECs and cytotoxicity to L3 and mf

**Fig 3:**
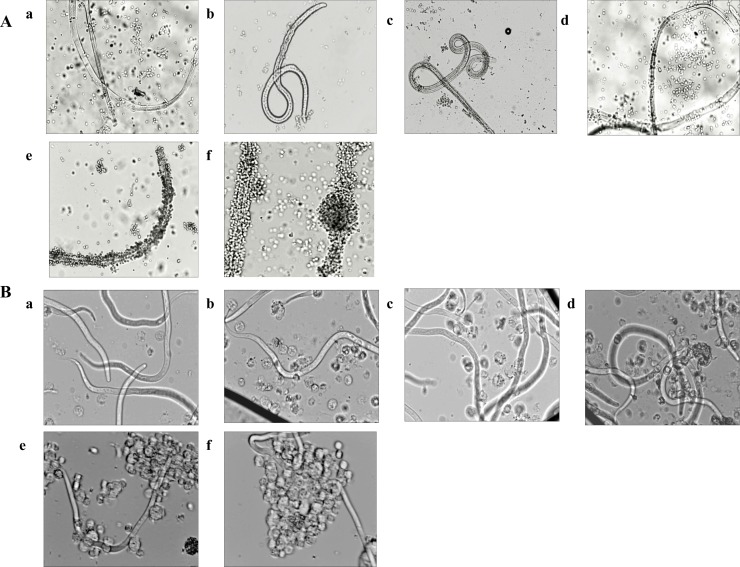
**Antibody-dependent cell-mediated adhesion and cytotoxicity (ADCC) to (A) L3 and (B) mf.** Adhesion of PECs on the surface of *B*. *malayi* L3 and mfwas observed after 48 h microscopically in the presence of sera of different groups (a) pcD (b) Adj (c) pcD+Adj and (d) Myo-pcD (e) Bm-Myo (f) Myo-pcD+Bm-Myo. Photographs were captured on phase contrast fluorescent microscope (Nikon, Japan) at 40X magnification.

Marked increase in cellular adherence was observed on the surface of L3 and mf in presence of Myo-pcD+Bm-Myo immunized sera (1:16 dilution) as compared to Bm-Myo or Myo-pcD. The heterologous group caused 75.0±0.63% and 71.67±0.97% cytotoxicity and death of L3 and mf respectively as compared to Bm-Myo (L3 = 57.3±0.65%; mf = 56.55±1.45%; P < 0.05) and Myo-pcD (L3 = 25.0±0.58%; mf = 30.54±2.11; P <0.001) (Table 1, Fig 3).

**Table 1:** Antibody-dependent cellular adherence and cytotoxicity (ADCC) to L3 using sera of immunized and control *M*. *coucha*.

Groups	No. of animals	Percentage cytotoxicity	
		Microfilariae	L3
**pcD**	**10**	**10.0±1.4**	**17.0±0.61**
**Adj**	**10**	**9.5±1.6**	**16.9±0.72**
**pcD+Adj**	**10**	**10.9±2.0**	**17.3±0.81**
**Myo-pcD**	**10**	30.54± 2.11*	**25.3±0.58**
**Bm-Myo**	**10**	56.55±1.45***	57.3±0.65***
**Myo-pcD+Bm-Myo**	**10**	71.67±0.97***	75.0±0.63***

ADCC assay was performed by incubating mice sera (n = 10) samples with 1×10^5^ normal PECs and 10 *B*. *malayi*L3 and 100 mf at 37°C for 48 h. Sera from animals in each group were used in duplicate for the assay. Statistical significance of the differences between mean values of immunized and control groups are shown as * P<0.05 and *** P<0.001.

* P<0.05, i.e., statistical significance

*** P<0.001, veryhigh significance

### Effect on microfilarial density

**Fig 4:**
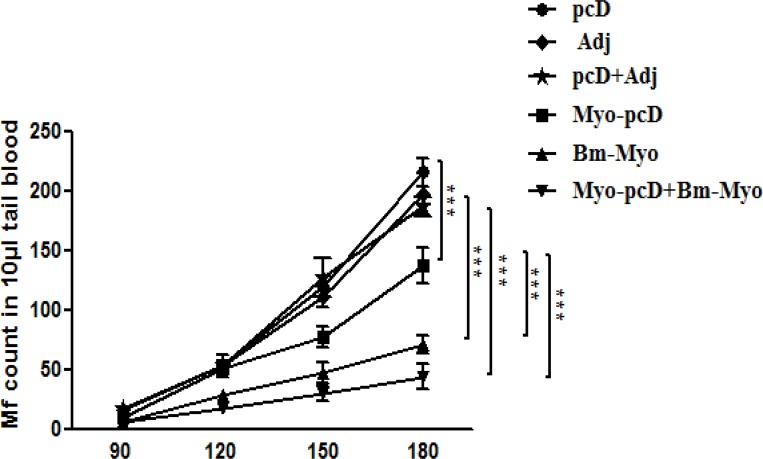
Microfilarial kinetics in different immunized group of *M*.*coucha*. Microfilaraemia in the tail blood of immunized and control groups of *M*. *Coucha* were determined after 90 days p.c L3. Micofilarial density was significantly reduced at 180 days in Myo-pcD+Bm-Myo compared to Bm-Myo(*P < 0.5) and Myo-pcD (***P < 0.001) and remained low throughout the observation period while all the control groups showed a sharp increasing trend in the following weeks. Myo-pcD+Bm-Myo, Bm-Myo and Myo-pcD groups exhibited 78.5±11.06%, 66.5±7.49%, 37.0±14.88% reduction in microfilarial burden compared to their respective control groups. Experiment was performed in duplicate and data of both were later merged as one. Each point represents a value of mean±SE of 10 animals and the statistical significance was indicated *P < 0.05, **P < 0.01, ***P < 0.001.

Mf start appearing in blood circulation after 3 months p.c, therefore, assessment of tail blood microfilaraemia in *M*. *coucha* was initiated on day 90 p.c and continued up to day 180 when all the animals were euthanized for the recovery of adult worms. There was a sharp increase in mf numbers in all the control groups while mf counts were significantly low in Myo-pcD+Bm-Myo group (P < 0.05–0.001) which was even lower than Bm-Myo and Myo-pcD groups (Fig 4). There was 78.5±11.06% reduced mf level in blood of Myo-pcD+Bm-Myo group while Bm-Myo and Myo-pcD groups showed 66.5±7.49% and 37.0±4.88% reduction respectively over respective controls.

### Myo-pcD+Bm-Myo vaccination causes significant reduction in *B*. *malayi* L3 establishment, adult worm recovery and female worm fecundity

**Fig 5:**
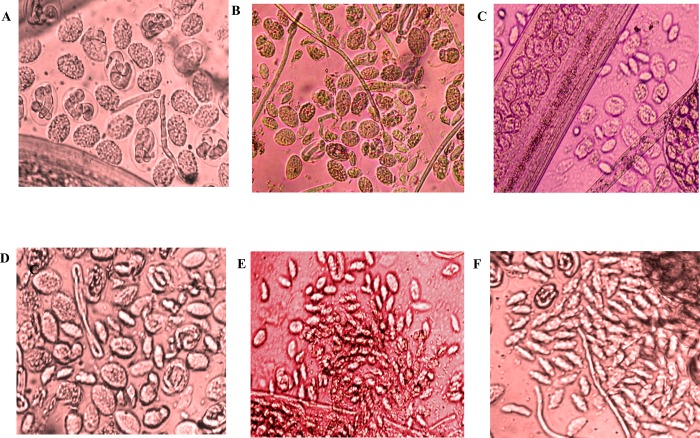
Intrauterine content of females recovered from control and immunized animals. Females from control groups **(A)**pcD**(B)**Adj**(C)**pcD+Adj were showed fertile eggs in uterine contained showing normal phenotype and developing stage. **(D)** Females from Myo-pcD group showed few degenerated eggs while the uteri of females recovered from **(E)**Bm-Myo and **(F)**Myo-pcD+Bm-Myo contained degenerated eggs.

All the immunized and control *M*. *coucha* were euthanized humanely on day 180 p.c and adult worms from heart, lung, testes and lymph nodes were recovered and counted. The number of worms and reductions in worm burden in different immunized groups is shown in Table 2. Vaccination with Myo-pcD+Bm-Myo led to noticeable reduction (75.3%) in adult worm recovery when compared with that of respective control groups and this was much higher than Bm-Myo (62.9%, P < 0.05) and Myo-pcD (30.5%, P < 0.001). In addition, a significant proportion of live female worms recovered from Myo-pcD+Bm-Myo vaccinated animals were rendered sterilized i.e. their uteri contained degenerated eggs, embryos and stretched mf (75.4±0.93%; P < 0.001) unlike Bm-Myo (55.7±1.12%; P < 0.001) or Myo-pcD (15.4±2.3%) groups where this effect was moderate (Table 2) (Fig 5).

**Table 2:** Adult worm recovery and sterilization in female worm recovered from *M*.*coucha*.

Groups	No. of animals	Adult worm counts in individual animal	Adult worms(Mean±SE)	Total adult worm recovery(Mean±SE)	Worm burden reductionover respective control (%)	Female wormsterilization (%)
**pcD**	**10**	**♀21,22,25,12,32,24,9,17,9,29**	**20.0±0.80**	**27.8±2.98**	**-**	**11.6±2.20**
		**♂9,7,11,9,6,11,5,6,4,10**	**7.8±1.22**			
**Adj**	**10**	**♀27,20,23,11,33,26,10,24,12,30**	**21.6±2.5**	**29.7±3.2**	**-**	**9.5±3.00**
		**♂9,1,15,8,5,14,4,5,9,11**	**8.10±1.41**			
**pcD+Adj**	**10**	**♀21,29,23,16,20,12,20,28,9,23**	**20.10±2.10**	**28.8±2.35**	**-**	**13.2±3.4**
		**♂8,6,12,9,1,11,7,10,8,15**	**8.7±1.19**			
**Myo-pcD**	**10**	**♀12,19,17,12,18,11,8,11,15**	**13.8±1.67**	**19.3±2.09**	30.5*	**15.4±2.3**
		**♂8,6,7,7,5,9,2,6,3,2**	**5.50±0.78**			
**Bm-Myo**	**10**	**♀5,8,15,10,11,7,5,9,10,3**	**8.30±1.11**	**11.0±1.10**	62.9***	55.7±1.12***
		**♂2,1,4,2,2,4,6,0,2,4**	**2.70±0.55**			
**Myo-pcD+Bm-Myo**	**10**	**♀4,3,11,9,8,12,4,10,7,8**	**7.60±0.97**	**7.3±0.87**	75.3 ***	75.4±0.93***
		**♂1,0,0,1,2,3,0,1,0,2**	**1.0±0.34**			

Significantly different from values obtained from control group

* P<0.05

***P< 0.001.

### Cytokine response in the immunized animals

**Fig 6:**
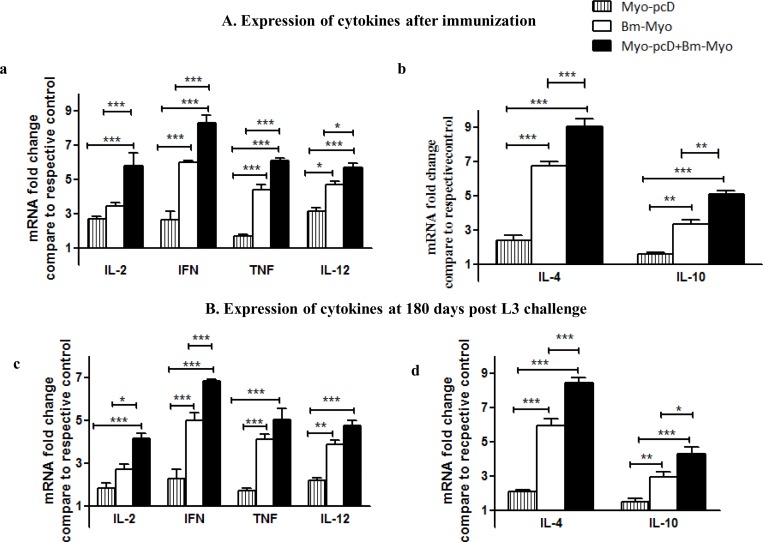
**Real time PCR analysis of mRNA expression levels of Pro-inflammatory and anti-inflammatory cytokines:** The expression of Th1 (IL-2, IFN-ƴ, IL-12 and TNF-α) and Th2 cytokines were analyzed **(A) after immunization (day 60)** and **(B) on day 180 post challenge**, in spleen of different groups of mice. Myo-pcD+Bm-Myo immunization up-regulated the expression of **(a)**Th1and **(b)**Th2 cytokines after immunization compared to Bm-Myo (P<0.05 –P< 0.001) and Myo-pcD (P<0.001). However, on day 180 p.c the expression of all the cytokines was slightly decreased but remained elevated compared to Myo-pcD (P < 0.001) while in comparison to Bm-Myo only IL-2, IFN-ƴ, IL-4 and IL-10 were found significant (P < 0.05—P< 0.001) and the level of IL-12 and TNF α were not significant. Values given are mean fold change as compared to their respective control group. Statistical significance between different groups is depicted as *P < 0.05; ** P < 0.01 and ***P < 0.001.

The mRNA expression of both Th1(IL-2, IFN-ƴ, TNF-α and IL-12) and Th2 cytokines (IL-4, IL-10) were determined by real time PCR at the end of immunization (day 60) and on day 180 post L3 challenge in spleen of all immunized and control groups of animals. The data revealed that following immunization, Myo-pcD+Bm-Myo significantly (P < 0.05–0.001) up-regulated the expression of both Th1 and Th2 cytokines as compared to Bm-Myo or Myo-pcD groups (Fig 6A). Though the cytokine expression slightly lowered at the time of autopsy however noticeable augmentation from controls could still be seen. The increase in TNF-α and IL-12 transcript levels was however not found significant when compared with Bm-Myo (Fig 6B).

## Discussion

Within the past few years, several antigens from *B*. *malayi* have been developed as recombinant proteins and also evaluated for their immunoprophylactic effectiveness [3, 27, 28] with varied success. Our earlier studies demonstrated moderate to high protective efficacy in some of the recombinant *B*. *malayi* and *Wolbachia* proteins [23,29–31] including *B*.*malayi* recombinant heavy chain myosin (Bm-Myo) in *M*. *coucha* and jird models [4]. Myosin is a structural protein existing mostly in muscle and is necessary for parasite mobility, although muscle proteins are evidently not surface antigens but in filariasis very small percentage of L3 normally develop in to adult parasites in the host as the majority get killed and this leads to release of tegumental and sub-tegumental proteins including muscle proteins and host may get exposed to these in this process. Immunization enhances it further as more and more L3 get killed with only a small proportion survives to develop as adult parasites. Filarial paramyosin and tropomyosin are also associated with muscle tissue which would also not normally be accessible to components of the immune system but the ultrastructural localization of tropomyosin in Onchocerca parasites revealed that in addition to the predicted localization to muscle tissue, reactivity within the cuticle was also observed [32–33]. We have earlier reported [4] the cellular adherence and cytotoxicity to L3 and mf in presence of immunized rodent serum. Furthermore, human bancroftian sera contained antibodies to *B*. *malayi* myosin which react strongly with the *B*. *malayi* lysate of all the major life stages including L3 in Western blots and also in ELISA. These findings suggest that myosin though remains in the muscles may also be either expressed on the parasite surface or released by the dead and disintegrating filariids that may render the host exposed to muscle protein.

It has long been observed that the acquired immunity to tegumental, sub-tegumental and internal antigens of Scistosome develops due to experience of parasitic antigen which gets speed up by treatment [34–37]. Most of the proteins identified by helminth proteomics have been found in ESP in previous studies, including cytoskeletal proteins (i.e. actin, tubulin, myosin, paramyosin, tropomyosin). The extracellular vesicles may also play an important role in communication between the parasite and the host [38]. The paramyosin of *T*. *solium* (TPmy) is present in the musculature but has also been found associated with the tegument of the parasite [39]. TPmy is synthesized by the tegumentary cytons and apparently released through the cyst tegument and TPmy can be collected in the culture medium in which *T*. *solium* cysts are maintained [40], suggesting that a similar release to the host tissues might occur *in vivo*.

Therefore, to further enhance the protective efficacy of earlier identified Bm-Myo, we cloned Bm-Myo in mammalian expression vector and used this plasmid construct (DNA vaccine) to immunize BALB/c mice with homologous DNA as well as DNA/protein prime boost which demonstrated better protection than when recombinant myosin was employed [21]. Findings also demonstrated that priming with DNA construct followed by protein boosters elicited more robust humoral and cellular immune responses and provided higher rate of protection against L3 in the peritoneal cavity of mice than when DNA vaccination was followed by DNA vaccine booster. These preliminary observations in mice suggested on the immunogenic nature of the Myo-pcD+Bm-Myo. Subsequently, the current study was undertaken in susceptible host, *M*. *coucha* that supports the full life cycle of *B*. *malayi* and therefore effect of immunization could be observed for several months on all the major life-stages (mf, L3 and adult) thereby mimicking the situation in humans. The results demonstrated that the heterologous prime-boost i.e. first two dose of DNA vaccine followed by two booster doses of recombinant protein provided best protection results. There was much higher production of specific IgG antibody by this regimen. In addition, Myo-pcD+Bm-Myo also provoked higher up-regulation in the levels of specific IgG1, IgG2a, IgG2b and IgG3 antibody isotypes and these levels were maintained throughout the observation period of 180 days unlike controls where patent microfilaraemia correlated with a dip in these parameters. IgG1 was more prominent followed by IgG2a reflecting generation of a mixed Th1/Th2 immune response marginally biased towards Th2 in contrast to recombinant Bm-Myo which caused higher production of IgG2a revealing more polarization towards Th1. It is documented that IgG1 and IgG2a antibodies offer protection against challenged filarial larvae post-irradiated L3 vaccination. Several other reports have suggested the major role of antibodies in ADCC mechanism and also protection against LF [41–43]. ADCC is one of the established immunological mechanisms operating against filarial parasites both *in vitro* and *in vivo* involving neutrophils, macrophages and eosinophils [41–43].The previous reports reveal that the antigens which induce antibodies that mediate cytotoxicity to parasite either *in vitro* or *in vivo* carried great vaccine potential and offered significant protection against filarial infection [6, 44]. The ADCC results showed maximum adherence of cells to the surface of L3 and mf especially in Myo-pcD+Bm-Myo immunized group and these animals developed very low density of mf in blood. One of the reasons could be the removal of mf via ADCC as demonstrated *in vitro* or due to low adult worm burden (~75.3% reduction) in Myo-pcD+Bm-Myo immunized group. Apart from reduction in parasite burden, large proportion of live female worms recovered from Myo-pcD+Bm-Myo immunized animals were sterile and had mostly degenerated eggs and mf in their uteri, thus sterility of these living female worms could also be one of the major reasons of low microfilarial level.

Myo-pcD+Bm-Myo appreciably up-regulated the levels of both Th1 (IL-2, IFN-ƴ, TNF-α, IL-12) and Th2 (IL-4, IL10) cytokines which remained elevated throughout the observation period. IFN-ƴ is known to stimulate the synthesis of IgG2a and has been suggested to play an essential role in worm clearance [45–46] along with increased levels of IL-2 and IFN-ƴ has been reported to be associated with immunity in human infections [47–48]. Moreover, IFN-ƴ and TNF-α activate macrophages to secrete ROS and NO that are crucial in parasite killing [49]. Similarly, IL-4 stimulates the production of IgG1 [50] and prevents filarial development in resistant mice and aids in the clearance of mf from the blood stream. IL-4 and IL-10 are the key cytokines of effector mechanism in murine filariasis [51] and filarial infections diminish both Th1 and Th2 pathways for survival, therefore, protective immunity required activation of both the arms of immune response [22].

In this present study, immunization with heterologous prime boost vaccination employing Bm-Myo protected *M*. *coucha* against the *B*. *malayi* challenge over a long period of six months. The long-term immunological memory was established as demonstrated by humoral and cellular response till the end of experiment and elevation of a mixed Th1 and Th2 type of immune response could be one of the reasons for bringing about the killing of large proportion of challenged L3 leading to reduced worm establishment and recovery from immunized *Mastomys*. The prime boost vaccination leads to first priming of the highly effective antigen-specific memory B-cells [52, 53] with DNA construct and further the enhancement of the cellular immune response by protein booster which may further stimulate memory B-cells to differentiate into antibody-secreting cells, resulting in production of high titers of antigen-specific antibodies [52]. Therefore, combination of Myo-pcD construct prime followed by a Bm-Myo boost appears to be an ideal approach that may overcome their respective limitations [54].
